# Recent Progress in the Detection of Bacteria Using Bacteriophages: A Review

**DOI:** 10.3390/v12080845

**Published:** 2020-08-03

**Authors:** Jan Paczesny, Łukasz Richter, Robert Hołyst

**Affiliations:** Institute of Physical Chemistry of the Polish Academy of Sciences, Kasprzaka 44/52, 01-224 Warsaw, Poland; lrichter@ichf.edu.pl

**Keywords:** bacteria detection, bacteriophages, phage-based sensors

## Abstract

Bacteria will likely become our most significant enemies of the 21st century, as we are approaching a post-antibiotic era. Bacteriophages, viruses that infect bacteria, allow us to fight infections caused by drug-resistant bacteria and create specific, cheap, and stable sensors for bacteria detection. Here, we summarize the recent developments in the field of phage-based methods for bacteria detection. We focus on works published after mid-2017. We underline the need for further advancements, especially related to lowering the detection (below 1 CFU/mL; CFU stands for colony forming units) and shortening the time of analysis (below one hour). From the application point of view, portable, cheap, and fast devices are needed, even at the expense of sensitivity.

## 1. Introduction

The growing problem of the appearance of multidrug-resistant bacteria might completely change our lifestyle. For the last 70 years, we have lived in a world where we have been able to kill bacteria on-demand with antibiotics. However, evolution, our lack of knowledge, and excessive use of antibiotics are slowly causing this situation to change. The gene mcr-1, responsible for resistance against colistin, an antibiotic often referred to as “drug of last resort”, was proved to have spread around the world [1] and was found in several bacteria (including *Escherichia*, *Salmonella*, *Klebsiella*, *Kluyvera*, *Citrobacter*, and *Cronobacter*) within a few years [2]. Bacteria could gain resistance to a given antibiotic within hours [3]. Recently, the first example of the adaptation of bacteria to physical factors (nanomechanical stress) was also reported [4]. Without proper means of detection, allowing for targeted use of drugs, we will be facing a scenario where small wounds could be a potential life threat, as they were before the discovery of penicillin.

**Figure 1:**
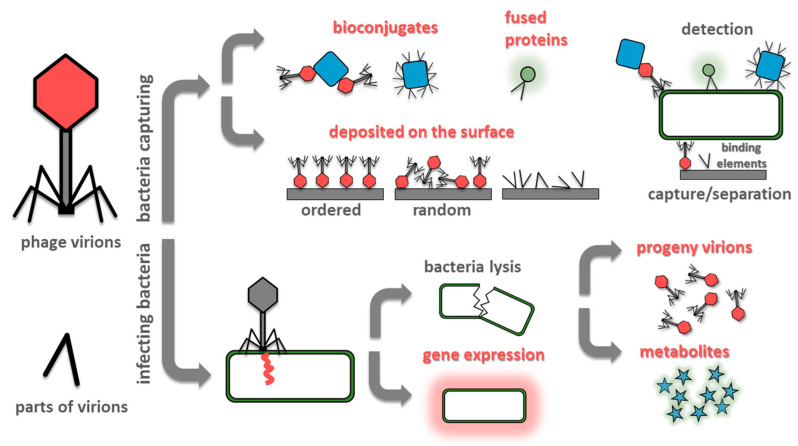
Summary of most often exploited designs of phage-based biosensors. Methods utilizing bacteria capturing (at the surface of the sensing elements or by phage-based probes) are fast. However, a single event generates a signal which is difficult to detect. Contrary, infecting bacteria and utilizing its molecular machinery to amplify the signal (by the generation of progeny virions, introduction reporter genes, or due to release of bacterial metabolites due to lysis) offers lower detection limits, but the procedures are lengthy.

The prevention of the spread of pandemics needs proper detection [5]. To monitor and implement appropriate control measures, the use of sensitive and specific diagnostic methods is paramount. Critical parameters of sensors for bacteria detection are the time of analysis, the limit of detection (LOD), sensitivity, and specificity. From the application point of view, also cost, portability, ease of use, operator hands-on time, and reliability are critical. Often one trait could be traded for another, e.g., the introduction of the pre-incubation step improves the limit of detection at the expense of time of analysis. Conventional microbiological methods for bacteria detection, based on culturing microorganisms, are cheap and selective but also time-consuming and laborious. Therefore, researchers are introducing new detection techniques. Over the past decades various detection methods have been developed including (but not limited to) nucleic acid-based sensors (DNA microarrays [6], polymerase chain reaction (PCR) and its derivatives, e.g., multiplex PCR or real-time PCR [7,8]); immune-based sensors (e.g., enzyme-linked immunosorbent assay [9]) and mass spectrometry sensors (especially MALDI-TOF-MS) [10]. All these techniques share significant drawbacks: they require equipment, trained users, and are costly. Therefore, biosensor-based methods are increasingly gaining acceptance. The most commonly used bio receptors are antibodies, enzymes, and nucleic acids [11]. However, bacteriophages became an exciting alternative in the field of rapid detection of bacteria [12,13]. The most commonly used designs for phage-based bacteria detection are shown in Figure 1.

Bacteriophages are viruses that infect bacteria. The average size of the virion (single phage) is around 50 nm to 200 nm. However, the largest bacteriophages are more than 400 nm in length [14]. The vast majority of all known bacteriophages (above 95%) belong to the order *Caudovirales*. They share a universal structure design, i.e., genetic information (dsDNA) is in a capsid, to which a tail with fibers is attached [15]. Much less common are filamentous (e.g., M13) or nearly spherical (isometric) phages (e.g., MS2).

Unlike antibodies, phages can be quickly and cheaply produced in large quantities and properly purified. By only infecting a bacteria solution, one can obtain a large number of progeny phages. Moreover, some phages are robust and retain their activity even after exposure to high temperatures [16], pH [17], and organic solvents [18,19]. Thanks to the abundance of different bacteriophage types, it is theoretically possible to design biosensors to detect almost every bacterial strain. The essential traits of phages are that they are efficient and specific against host bacteria [20,21]. Only recognition of a proper and viable host assures the multiplication of virions and completion of the life cycle. Thus, the utilization of phages allows for the distinction between live and dead cells, a common problem in other methods. One needs to acknowledge that phages might adsorb to the surface of the dead bacterium, thus affecting the methods relying purely on the detection of capturing events [22]. With the completion of life cycles, phages undergo evolution, so they are always up to date in the arms race against bacteria [23]. For instance, not long after discovering CRISPR [24], anti-CRISPR mechanisms were also found [25]. In general, phages are great candidates as biorecognition elements in biosensors and other assays.

## 2. Whole Phage-Based Bacteria Sensing

The most straightforward design of phage-based biosensors for bacteria detection utilizes whole virions as sensing elements. The majority of known phages belong to *Caudovirales* order. It gives promise for the possibility of simplifying the preparation process to utilize various phages for sensors preparation. Such an approach dramatically expands the potential applicability of new solutions allowing for the detection of a variety of target bacteria.

Another significant advantage of phage-based biosensors is the possibility to isolate phages specific against any target bacteria quickly and cheaply. There is even no need to identify isolates. Such an approach was used recently in several studies, and phages, which hosts are bacteria of interest, were isolated from hospital sewage water [26], and environmental samples [27,28,29].

The critical decision in phage selection is the choice between temperate and lytic phages. For instance, lytic phages are a must when the release of progeny virions or bacterial metabolites are to be detected. However, the utilization of lytic phages usually limits the incubation time to below one hour. Prolonged incubation results in lysis of cells captured early. Such time constraints restrict the possible number of captured bacteria in case of methods requiring proximity between cells and surface to generate an analytical signal.

### 2.1. Bacteriophages Deposited on Solid Substrates

Usually, virions (viral particles) are deposited at the surface. The role of the substrate in nanotechnology is usually to provide support and increase the robustness of functional material. In the case of sensors, the surface also often takes part in the sensing process being a part of a transducer. The transducer is an element of sensor, which generates a measurable signal upon capturing target bacteria. Three main designs have been explored where bacteriophages are deposited onto a solid substrate. One is based on electrochemical methods, with phages deposited on the electrodes. The second utilizes magnetoelastic sensors, where a change of mass upon bacteria capture changes the amplitude of vibrations. The third one comes from the surface-enhanced Raman spectroscopy, where excited plasmons within the substrate allow for enhancement of the intensity of the recorded spectra.

In electrochemical methods, the electric signal changes upon capturing of bacteria by virions deposited at the electrodes. There is an increasing number of published works utilizing the electrochemical approach to detect bacteria [26,30,31,32,33,34,35,36]. Electrochemistry offers good sensitivity, low-cost analysis, and allows for miniaturization. Also, the signal in the form of electric current or voltage is easy to process. The development of bacteriophage-based electrochemical methods for bacteria detections appears in various review articles published recently [37,38,39].

Sedki et al. [35] described setup utilizing M13 immobilized on the electrodes, by chemical methods, for electrochemical impedance spectroscopy. It allowed for the detection of coliforms with LOD of around 14 CFU/mL (CFU stands for colony forming units) within 30 min. In this example, a single phage allows for the detection of multiple strains of *E. coli*, while not responding to non-*E. coli* bacteria. Moon et al. have recently published a detailed review of M13-based biosensors [40].

In another interesting example, Yue et al. [41] reported a label-free electrochemiluminescent biosensor capable of detecting *Pseudomonas aeruginosa* with LOD of 56 CFU/mL within 30 min. The authors used the carboxyl graphene-PaP1 composite, acting a sensing element on the glassy carbon electrode.

Magnetoelastic sensors are usually ribbon-like strips of amorphous ferromagnetic alloys. They vibrate under magnetic excitation. Mechanical vibrations generate secondary magnetic flux that can be detected remotely. The deposition of analyte on the surface, covered with the sensing layer, changes the amplitude of the vibrations providing the analytical signal. Hiremath et al., 2015 [42] reported a sensor for the detection of methicillin-resistant *Staphylococcus aureus* with a limit of 3 × 10^3^ CFU/mL within 30 min. In 2017 they confirmed that MRSA was detected specifically and selectively even in the presence of other competing bacteria [43]. Chen et al. [44], and Mack et al. [45] showed other exciting applications. Both papers describe the detection of *Salmonella* (*S. enterica* and *S. typhimurium*, respectively), at the surface of food products (chicken and lettuce, respectively). In both cases, the magnetoelastic strip was pressed against the sample to be analyzed.

Surface-enhanced Raman spectroscopy (SERS) is a technique utilizing localized surface plasmon resonance of metal surfaces to obtain ultrahigh enhancement of Raman scattering. It allows for an increment of the intensity of recorded spectra by many orders of magnitude. For instance, commercially available substrates (e.g., *SERSitive*) offer enhancement factors in the range of 10^7^ to 10^8^ for some analytes [46]. Such properties allow for the detection of analyte at an ultra-low concentration, reaching the single molecules level [47]; however, in the case of larger analytes, such as bacteria, the situation is more complicated. The potential of SERS for bacteria detection was first proved for cells deposited directly on the SERS-active substrate [48]. One of the first examples of successful phage utilization as a sensing layer in SERS was demonstrated by Srivastava et al. in 2015 [49]. They used thin silver films on a silicon platform along with T4 phages. The reported limit of detection of *E. coli* was 1.5 × 10^2^ CFU/mL. Recently, Rippa et al. [50] developed a substrate made of plasmonic nanocavities, with a layer of immobilized bacteriophages. The authors suggested that the proposed system constitutes a novel solution for the specific detection of different species of bacteria. The same group successfully used other metastructures, appropriately functionalized with *Tbilisi* bacteriophages, for Brucella’s SERS-based detection. The authors performed measurements within only one hour and at the single-cell level, with bacteria deposited from a suspension of concentration higher than 10^4^ CFU/mL [51]. Lai et al. [52] achieved similar LOD using principal component analysis (PCA) to process obtained SERS spectra in the detection of *Bacillus* spp. using gamma phages.

#### Oriented Layers of Bacteriophages

**Figure 2:**
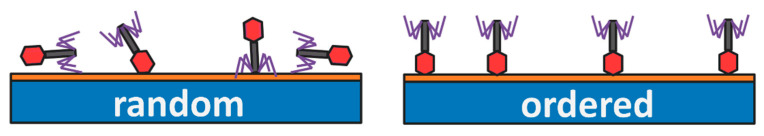
Upon deposition of phages at the solid surface, the entropy favors the random orientation of virions, which restrict the possibility of interactions between fibers (marked in violet) with target bacteria. Proper orientation of virions allows for more receptor binding proteins (RBPs) to participate in the sensing process.

Until recently, the process of deposition of phages onto the solid substrate was poorly controlled [53,54]. This process did not affect filamentous or isometric phages but appeared crucial in tailed phages, constituting the majority of all known phages. In the case of deposition of tailed phages, entropy favors the alignment in which the long axis of the virion is parallel to the solid substrate (e.g., transducer). As this process is random, some virions may orient parallel to fibers attached to the surface. Such orientations restrict the possibility of interactions between fibers and receptor binding proteins (RBPs) with target bacteria (Figure 2). Only orientation in which the long axis of the virions is vertical to the surface, and the tails are facing upward, assures that most RBPs are involved in the bacteria detection process. Phages that infect a bacterium are oriented nearly perpendicular to the surface of the bacterium cell wall. Phages are probing a bacterium surface with fibers oriented under a large angle, typically >45 degrees to the surface plane. This probing is reversible.

Researchers strive to achieve denser coverage of the surface. For instance, in recent work, Farooq showed the high-density phage particles immobilization for ultra-sensitive and selective detection of *Staphylococcus aureus* [26]. Griffith’s group published first attempts to increase the number of available RBPs not by increasing the number of randomly oriented virions, but through proper orientation at the beginning of 2010s [55,56]. Tolba et al. [55] used recombinant bacteriophages, which had the outer capsid protein gene of T4 fused with genes coding specific binding proteins (biotin or cellulose-binding module). These binding proteins were present only at the surface of capsids. Upon binding, tails and fibers remained exposed and available for bacteria.

Nevertheless, it was not until 2016 that the researchers quantified the effect of proper orientation. Richter et al. [57] exploited permanent dipole moment of virions to orient T4 phages along the electric field lines. The number of deposited phages did not change significantly upon the application of the electric field. However, the increase in the number of captured *E. coli* cells was fourfold compared to the randomly oriented layer of phages. The orientation of phages in the constant electric field was, in fact, an example of charge driven assembly. A similar approach was utilized by Anany et al. [56] who used charged cellulose membranes, or Zhou et al. [32], who reported a carbon nanotube (CNT)-based impedimetric biosensor, in which T2 phages were oriented upon application of constant potential. Recently, Imai et al. [46] claimed to utilize core−shell nanoparticles with the surface charge to orient phages S13 properly. Results reported by Liana et al. [58] suggest that there is another layer of complexity in such a seemingly simple system. The authors compared amino- (positive charge in neutral pH) and carboxylic- (negative charge in neutral pH) functionalized surfaces of indium tin oxide (ITO). It was found that more T4 virions were adsorbed on negatively charged planar ITO, whereas in the case of particulate ITO, better coverage was achieved for positively charged particles. The authors explained it in terms of variations of time available to bind phages on planar (longer time required) versus particulate (shorter search time) ITO [58]. Anyhow, Bone et al. [59], again proved that chemisorption allowed for much higher surface coverages.

When charged surface (let it be due to chemical modification or application of constant electric field) is in the electrolyte (e.g., buffer), the electrical double layer is created. It screens the electric field, restricting its effective range to Debye length. The value of Debye length in standard buffer varies from below one to dozens of nm, with values in the range of few nm being most common. This length is much smaller than the size of the virion. To overcome such screening, Richter et al. [60] utilized alternating electric field (i.e., varying in time according to programmed time traces) to promote the vertical arrangement of virions. They used a trapezoidal waveform with interpulse periods with no potential applied. Periods without applied electric field allowed for relaxation of the electrical double layer. Such an approach resulted in a tenfold increase in the number of captured bacteria comparing to randomly oriented phages. When combined with chemical immobilization of phages, the increase was around 62-fold. It allowed for obtaining a limit of detection in a range of 10^2^ CFU/mL by using just 15 min incubation time. Very recently, Xu et al. [36] systematically studied the influence of Debye length and concentrations of phage suspensions on the performance of a T4 bacteriophage-based micro electrochemical sensor, with a sensing layer ordered in the alternating electric field. The applied conditions were similar, as in the case of Richter and coworkers [60]. When the Debye length was comparable to the phage size, the capture efficiency attains the maximum value. The obtained limit of detection was 14 ± 5 CFU/mL.

Such an approach of deliberate design of functional biomaterials is gaining importance. An example of a recent review on controlling the self-assembly of biomolecules by adjusting internal interactions of interactions and due to external stimulation was published by Wang et al. [61]. There are many more possible applications of such an approach. For example, the electric field was used to assemble M13 phages into colored films. Due to the facile functionalization of virions, it might be used to prepare the nanodevices [62]. In work by Tronolone and coworkers [62] the application of an AC electric field to an evaporating droplet of M13 bacteriophage caused the motion of the meniscus of the droplet. During the movement of the meniscus, M13 virions adsorbed to the surface and assembled into smectic helicoidal nanofilament. The formation of such bundles manifested as rings of color bands.

### 2.2. Bacteriophage Based Bioconjugates

The intrinsic disadvantage of the design in which the sensing layer is deposited at the surface is a relatively low probability of bacteria being in the vicinity of the immobilized phages. In the case of small molecules, even low concentrations translate to a reasonably large number of objects to be detected (e.g., for picomolar concentration, which is usually considered low, the number of molecules is around 10^8^ per mL). However, in the case of bacteria, the aim is to detect a few bacteria in one mL or better. A low concentration of bacteria corresponds to a significantly lower number of detectable events, i.e., capturing bacteria by phages, in a given time.

One of the ways to counteract this is to incorporate the proper deposition technique. Very recently, Richter et al. [63] showed the potential of using the electric field to increase the number of analyzed objects directly at the surface. Another possibility is to increase the available surface area by using nanoparticles conjugated with bacteriophages. For instance, proof-of-concept studies showing the application of conjugates composed of gold nanoparticles and P9b phage displaying specific peptide binding to *Pseudomonas aeruginosa* for SERS detection was reported in 2020 [64]. In another example, gold nanoparticles were used to prepare a colorimetric sensor. Due to the alteration of surface plasmon resonance properties, suspension of gold nanoparticles changes its color upon aggregation. Peng and Chen [65] used chemically modified and genetically engineered M13 phages with exposed SH groups and displayed receptors against target bacteria (two strains of *E. coli*, *P. aeruginosa*, *Vibrio cholerae*, and two strains of the plant pathogen *Xanthomonas campestris*). First, modified M13 phages were added to the sample. After centrifugation, phages were present in pellets only if attached to target bacteria. The pellet was resuspended in buffer containing gold nanoparticles (AuNPs), which attached to SH groups at the surface of virions. This process resulted in aggregation and, finally, in a color change. The assay allowed for the detection of around 10^2^ cells (in 1 mL of the sample) within a 30-min procedure [65]. Another example utilized bacteriophages immobilized at the surface of the core-shell SiO_2_@AuNP nanoparticles for darkfield microscopic detection. Numerous conjugates attached to the target cell resulting in apparent aggregation causing strong light scattering. The authors reported *Staphylococcus aureus*’ detection within 15 to 20 min with a detection limit of 8 × 10^4^ CFU/mL [66].

Janczuk et al. [67] used magnetic and fluorescent particles to create phage-based bioconjugates used as flow cytometry probes. Reported LOD of *E. coli* using T4 phage bioconjugates was in the range of 10^4^ CFU/mL, and the incubation time was 15 min. Bacteriophage modified magnetic particles were also used for isolation and separation [68]. Isolation and separation are often combined with the detection of bacteria using an auxiliary detection method, e.g., immunoassay. Yan et al. [27] used such an approach that allowed for LOD of around 9 × 10^3^ CFU/mL in the detection of *S. aureus* in apple juice within 90 min without any pre-enrichment. Liana and coworkers [69] used bacteriophage conjugated Fe_3_O_4_ particles for the rapid capturing and isolation of *E. coli*. The authors focused on the optimal parameters of the operation of such a probe. They found that bacteria capturing occurred only at 37 °C in tryptone-containing media.

Bhardwaj and coworkers [28,29] used metal-organic frameworks (MOF) crystallites as phage carriers. First, they used IRMOF-3 (Zn_4_O(NH_2_-BDC)_3_) (NH_2_-BDC = 2-aminoterephthalic acid) conjugated with isolated lytic bacteriophage as a fluorescence probe. Upon binding, bacteria geometrically concealed MOF particles. The excitation energy which reached MOF particles was thus restricted, and a loss in the fluorescence intensity was recorded. The loss in fluorescence correlated with an increasing number of bacteria. The reported LOD of *Staphylococcus arlettae* was around 10^2^ CFU/mL [28]. Later, the same group used NH_2_-MIL-53(Fe), which is similar to IRMOF-3. NH_2_-MIL-53 (Fe) is iron- rather than zinc-based MOF but utilizes the same organic linker (NH_2_-BDC). Again, the fluorescence of the probe decreased with an increase in the bacteria concentration. TheLOD) of *S. aureus* was 31 CFU/mL, and the assay time was around 20 min [29].

Li et al. [34] presented a fascinating strategy for bacteria detection. They used complex organic-inorganic particles, designed to support the cascade of three electrochemical reactions, which acted as an amplifier. Cu_3_(PO_4_)_2_ nanoflowers were first loaded with glucose oxidase, horseradish peroxidase, and thionine. Next, gold nanoparticles were incorporated into such loaded nanoflowers. T4 phages were attached to gold via nonspecific bonding. The bacteria detection procedure was composed of multiple steps. First bacteria were immobilized at the surface of the electrode by antibodies or antimicrobial peptide magainin I. Loaded nanoflowers attached to such deposited cells upon phage binding to the surface of target bacteria. As glucose oxidase, horseradish peroxidase, and thionine appeared in the vicinity of the electrodes, electrochemical reactions occurred. First, glucose present in the buffer was oxidized. This generated hydrogen peroxide, in the second step, was used to oxidize thionine, which was regenerated at the electrode resulting in signal generation. The current increase was measured employing differential pulse voltammetry. Achieved LOD was in the range of 1 CFU/mL achieved within 140 min.

### 2.3. Genetically Modified Phages

Instead of conjugating phages to additional functional particles or molecules, it is also possible to introduce genetic modification into the phage genome to become both sensing and signal-generating elements. The most straightforward approach is to incorporate gene coding fluorescent protein or enzymes, generating easy-to-detect products. Upon infection, this gene is expressed inside the host cell, which allows for its detection. There are, however, some drawbacks of genetically modified phages. First, it is challenging to obtain modified phages, and the modification process and its optimization need to be repeated for each new target bacteria of choice. Secondly, modified phages are often less infectious [70]. Finally, the environmental risks of the unpredictable effects of modified phages on the biosphere are still not assessed [71]. A very recent review of engineered bacteriophages’ practical applications was published by Pizarro-Bauerle and Ando in 2020 [72]. The authors showed examples of the utilization of genetically modified bacteriophages in phage therapies, medicine, animal industry, and agriculture as sources of new antimicrobials, biocontrol, and genetic engineering tools. Here, we focus only on biosensing.

Wisuthiphaet and coworkers [73] showed the utilization of the T7-ALP phage, with introduced gene coding alkaline phosphatase, which was overexpressed upon infection of *E. coli*. Fluorescent substrate for alkaline phosphatase activity coupled with fluorescence imaging and image analysis was used for the detection of bacteria. The procedure took six hours and allowed for LOD of around 10^2^ bacteria per gram of model beverage samples.

Kretzer et al. [74] combined magnetoseparation, harnessing cell wall-binding domains from *Listeria* phage endolysins, with A511::luxAB bioluminescent reporter phage assay. Thus they combined two phage-based protocols into one. The final protocol gave LOD of around 10^2^ cells per mL of several *Listeria* strains within six hours.

Nugen group showed detection of *E. coli* with LOD of around 5 × 10^2^ CFU/mL after two hours incubation with T7 containing NanoLuc luciferase expression cassette. The method required the addition of a substrate for signal generation. Modified phages were prepared by synthetic biology strategy to engineer phages using a simple in vitro method [75]. The method used PCR fragments and in vitro DNA assembly followed by rebooting through transforming into host bacteria and not DNA assembly in yeast. The procedure resulted in the relatively simple and fast preparation of specific phages needed for detecting target bacteria in various applications.

Hinkley et al. [76,77,78] aimed to achieve a limit of detection sufficient for the analysis of drinking water. The standard in the United States mandates a zero-tolerance of generic *E. coli* in 100 mL of water. Their approach was to filter 100 mL of water sample on the cellulose filter. After 8 to 12 h of incubation necessary for colony development, two modified T7 phages with a reporter gene (luciferase or alkaline phosphatase) fused to genes for carbohydrate-binding modules (CBM) specific to cellulose were added and incubated for 1.5 h. In the final step, the enzymatic substrates were added, which allowed for the visualization of colonies. Overall, the process was around two times shorter comparing to standard procedure (10 h versus 24 h) and allowed for the limit of detection of around 1 CFU per 100 mL [76]. To shorten the required time of analysis, the same group proposed a T7 phage with the NanoLuc reporter gene fused again to CBM. First, the water sample was supplemented with concentrated growth media to allow the resuscitation of *E. coli*. After 60 min, genetically modified phages and microcellulose were added. During a further 90 min of incubation, the expression of NanoLuc-CBM occurred. The protein bounded to cellulose was collected by centrifugation. The luminescence was measured after the addition of the NanoGlo substrate to the sample. This approach offered a limit of detection better than 10 CFU/mL. However, the authors claimed that future improvements in the capture efficiency of the fusion reporter protein to cellulose should limit the detection of below 10 CFU per 100 mL [77]. Wisuthiphaet suggested that such limits of detection of these two setups could not be achieved in complex matrices due to background signals [73]. Finally, in 2020, Nugen’s group reported a syringe-based biosensor using the same engineered T7 phage containing the NanoLuc-CBM cassette, which allows for the limit of detection of around 20 CFU of *E. coli* in 100 mL of drinking water within 5 h [78].

Some systems do not require any external substrate to allow for detection. For instance, Vinay and coworkers [79] showed detection of *E. coli* and *Salmonella enterica* Typhimurium using HK620 and P22 phages, respectively, with introduced the GFP gene. Utilization of flow cytometry allowed for LOD as low as 10 cells/mL in seawater after one hour of incubation. The same group also reported the utilization of two engineered phages, namely HK620 and HK97. They had an entire luxCDABE operon that encodes luciferase and the substrate generating system. The reported LOD was in a range of 10^4^ bacteria/mL, but the main goal was to incorporate this probe into COMBITOX. This instrument aimed to accommodate several biodetector systems to detect pollutants such as bacteria, toxins, and heavy metals [80]. Much better limit of detection was reported by Kim et al. [81], who showed utilization of phiV10 phages also with luxCDABE operon. The authors showed the detection of *Escherichia coli* O157:H7 with LOD of around 1 CFU/mL in a pure culture within 40 min after 5 h of preincubation. In artificially contaminated romaine lettuce, apple juice, and ground beef, phiV10lux allowed for detection limits of around 10 CFU/cm^2^, 13 CFU/mL, and 17 CFU/g, respectively.

Wang et al. [33] showed the electrochemical detection of *E. coli* upon completion of the lytic cycle of T7 phages containing lacZ operon encoding β-galactosidase. The endogenous and phage induced β-galactosidase was detected using differential pulse voltammetry method with 4-aminophenyl-β-galactopyranoside as a substrate. Achieved limit of detection was in the range of 10^2^ CFU/mL within 7 h.

Rondón and coworkers [82] demonstrated the utilization of reporter phages in real-world applications. The authors used *mCherry_bomb_*φ phage for the detection of *Mycobacterium* spp. and phenotypic determination of rifampicin resistance. The study was performed on samples collected from 283 adult presumptive tuberculosis patients. However, the protocol took as much as three to five days.

### 2.4. Phage Amplification

In the bacteria sensing methods mentioned above, bacteriophages acted as specific binding agents. Such an approach is similar to antibody-based detection, but antibodies are much more expensive, difficult to prepare, and less stable than phages. Both phage- and immune-based methods are limited by the transducers’ performance, especially at an ultralow concentration of target bacteria. However, bacteriophages also offer a “built-in” amplification system. Instead of detecting capturing events, it is possible to search for progeny virions released from the host cell upon completion of the phage life cycle. A large number of released virions offers a few tens up to a thousand-fold multiplication of the number of objects to be detected. However, there are some disadvantages to such an approach. First, such methods require virulent and not temperate phages. Secondly, progeny phages will likely not be produced if there is already a prophage incorporated in the host’s genetic material. Thirdly, bacteria possess mechanisms preventing phage infections, e.g., CRISPR-Cas [24].

The most often used is a combination of phage amplification and detection of progeny virions employing the PCR technique. Several reports utilizing such an approach were published, with time and limits of detection varying strongly. For instance, Luo et al. [83] showed the detection of *Acinetobacter baumannii* in serum using p53 phages allowing for LOD in the range of 10^2^ CFU/mL within 4 h. Later they improved the method and achieved LOD of 10 CFU/mL in sputum samples within 6 h [84]. Garrido-Maestu et al. [85] showed the detection of 8 CFU of *Salmonella* Enteritidis in 25 g of chicken samples within 10 h. Extending the time of the analysis allowed Sergueev and coworkers [86] to achieve LOD of around 1 CFU/mL of *Brucella abortus* within 72 h in mixed cultures and blood samples. The most inspiring example was published by Anany et al. [87], who developed a phage-based paper dipstick biosensor to detect various foodborne pathogens in food matrices. They used piezoelectric inkjet printing to prepare phage-based bioactive papers that actively lysed their target bacteria. In combination with quantitative real-time PCR, this allowed for a limit of 10 to 50 CFU/mL in the number of various samples with a total assay time of 8 h.

Mido et al. [88] coupled amplification of phages with immunoassay. Progeny MS2 phages were captured by antibodies coupled on the surface of magnetic beads. Upon the addition of the detector antibody (also binding to MS2) the fluorescence was measured. The fluorescence allowed for the limit of detection of around 10^2^ cells/mL of live *E. coli* cells after a 3 h incubation.

A much more straightforward method of detection of progeny virions is titration using the plaque counting method. In this method, phages are deposited onto the agar plate inoculated with bacteria. In the place where virion is present, bacteria are lysed. Visible holes, plaques, appeared in the bacteria layer. Said et al. [89] used this approach to monitor the activity of a foodborne and waterborne pathogenic bacterium, *Salmonella typhi*, under starvation conditions. Phage infectivity rate was used to detect active bacteria that are not detectable by conventional methods, i.e., VBNC (viable but nonculturable) cells. In this approach, free phage concentration after incubation with samples containing VBNC cells (P_n_) was compared to the initial phage titer (P_0_). Analysis of kinetic parameters, e.g., the phage amplification rate (P_n_/P_0_) allows detecting the presence of active bacteria underestimated by using conventional methods.

### 2.5. Detection of Bacterial Metabolites

Upon completion of the lytic cycle, not only progeny virions are released, but also the content of the cell, including essential biomarkers. These biomarkers are typical for a variety of bacteria. However, the utilization of phages allows for the specificity of such approaches. For instance, Tilton et al. [90] reported a biosensor platform based on T7 bacteriophage, which mediated specific lysis of target bacteria and the release of β-galactopyranoside. β-galactopyranoside catalyzed the cleavage of the substrate resorufin β-D-galactopyranoside. The cleavage resulted in the formation of highly fluorescent resorufin. The fluorescent signal was detected using an epifluorescence system. Utilization of nanophotonic substrate allowed for the limit of detection of 10 CFU/mL of *E. coli* in simulated spinach wash water within 8 h.

He et al. [91] proposed a *Pseudomonas aeruginosa* detection setup combining magnetoseparation, phage amplification, and detection of intracellular adenosine triphosphate upon cell lysis and release of progeny virions. PAP1 phage was isolated from hospital sewage and conjugated with magnetic beads. Firefly luciferase-adenosine triphosphate bioluminescence system was used to determine the concentration of *P. aeruginosa*. Reported LOD was 2 × 10^2^ CFU/mL obtained within 2 h.

## 3. Parts of Phages Used for Detection

The procedure of isolating and amplifying phages against target bacteria from, for example, sewage water is relatively simple. One does not even need to identify the specific phage. However, there are some fundamental issues with biosensors utilizing whole virions as sensing elements. First, virions might be relatively large, and thus there is a limit of miniaturization. For instance, for magnetophoretic separation, magnetic particles conjugates to virions need to be in at least a sub-micrometer scale. Otherwise, the force exerted by the external magnet is not enough to drag the conjugate or the conjugate with the attached bacteria.

Moreover, in many analytical techniques (e.g., surface plasmon resonance), binding events need to happen within a given distance from the transducer’s surface. The relatively large size of phages might result in too long distance between the surface and the target analyte, thus hindering the analytical signal generation. The second disadvantage is related to the orientation of phages, which we did cover in Section 2.1. In short, parts of the virions that do not take part in bacteria capturing might create a steric hindrance for RBPs. Finally, the majority of bacteriophages ultimately cause lysis of the bacterial cells. It prohibits a more prolonged analysis in the case of samples of low bacteria concentration. It might happen that while waiting for other bacteria to attach to the sensor, the first bound bacterial cell is already destroyed.

The solution might be the utilization of parts of phages for the preparation of biosensors. For instance, He et al. [92] used recombinant tail fiber protein (P069), expressed in *E. coli*, for the detection of *P. aeruginosa*. The authors used two different approaches to detection. First, the recombinant protein was conjugated to magnetic beads. Such beads were added to the sample, and target bacteria were magnetically separated. After washing, the cells were disrupted, and the concentration of ATP was evaluated using the bioluminescence method. The second approach was based on P069 deposited onto the solid substrate. After capturing bacteria, fluorescently labeled P069 was added, allowing for fluorescence detection. The observed limits of detection were 6.7 × 10^2^ CFU/mL and 1.7 × 10^2^ CFU/mL for bioluminescent and fluorescent methods, respectively, within 60 to 80 min.

Similarly, Wang et al. [93] utilized bacteriophage cell-binding domain (CBD) and green fluorescent protein fused to CBD for a broad-spectrum recognition of methicillin-resistant *Staphylococcus aureus* strains. First, CBD conjugated magnetic beads were used to separate target cells, which were later detected employing flow cytometry upon the incubation of CBD-GFP protein. The protocol allowed for LOD of around 40 CFU/mL, and the procedure took around 1 h. Gomez-Torres et al. [94] also used CBD-GFP protein and compared it with GFP-CTP1L. CTP1L is a bacteriophage endolysin active against *Clostridium tyrobutyricum*. The authors were able to detect 17 of 20 *Clostridium* strains, also in the form of clostridial spores. GFP-CTP1L and GFP-CBD were used as biomarkers for the detection of *Clostridium* spores in milk.

An interesting example was reported by Liu et al. [95], who used bovine serum albumin-templated Co_3_O_4_ magnetic nanozyme (Co_3_O_4_ MNE) conjugated to *S. aureus*-specific fusion-pVIII (Co_3_O_4_ MNE@fusion-pVIII). First, the unbound triple-functional conjugates were magnetically separated from Co_3_O_4_ MNE@fusion-pVIII@*S. aureus* complexes. Next, peroxidase mimetics activity of the Co_3_O_4_ MNE was exploited to detect the target bacteria with a limit of around 8 CFU/mL.

Here, we review recent developments in bacteriophage-based methods for bacteria detection without omitting the fundamentals. We focus on reports published in 2017 and later, i.e., not covered by our last review [13]. Other, most recent, and general reviews on the topic were also published around that time [11,12], and thus we believe it is justified to provide an update. The summary of the performance of the phage-based biosensors is given in Table 1.

## 4. Concluding Remarks

**Figure 3:**
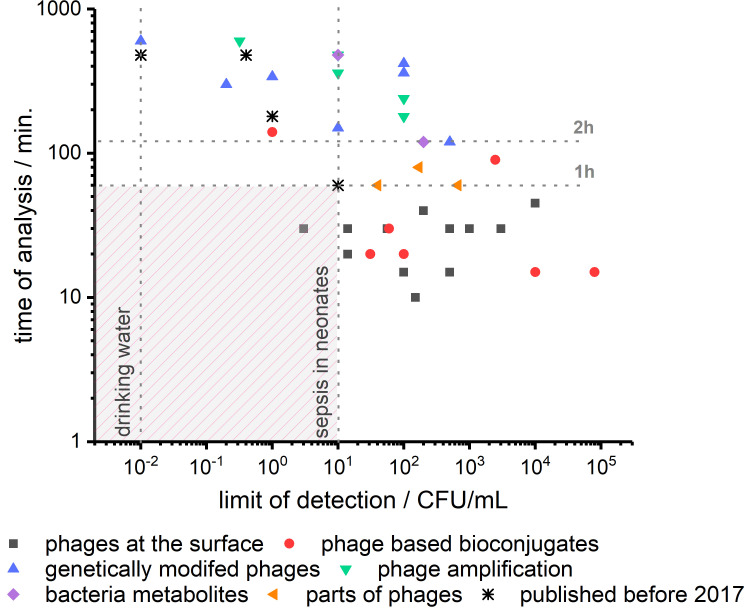
The relation between time of analysis and limit of detection of phage-based biosensors reported in scientific publications shown in Table 1. Recent advances in phage-based biosensors’ development bring us closer to fast and sensitive methods for bacteria detection. To achieve detection in the range below 10 CFU/mL in time below 1 h is still a crucial challenge.

In Figure 3, we depict the performance of recently reported phage-based biosensors in terms of limit of detection and time of analysis. We marked 10 CFU/mL as the limiting concentration of bacteria in blood in the case of sepsis in neonates. The second vertical line corresponds to 1 CFU per 100 mL of water, which is needed to analyze drinking water. A horizontal line marks another critical parameter at the time of analysis of 1 h. It is time for medical doctors to wait for the information on which bacteria are causing sepsis before the administration of wide-spectrum antibiotics [101]. Targeted treatment is, of course, beneficial for patients, but it needs to be introduced before the bacteria can cause severe damage. As it is clear from Figure 3, phage-based biosensors have just begun to enter the zone of fast and sensitive detection of bacteria.

There are two main approaches to achieve this. The first is to increase the sensitivity of phage-based bioconjugates, layered sensors, and methods utilizing parts of phages without additional preincubation steps. These methods usually are relatively fast, as the event to be detected is bacteria capture. The capture usually takes only minutes. However, it is challenging to detect capturing events, especially when the concentration of bacteria is low. In such a case, not only the number of events to be detected is low, but also the time of search might be extended. The second approach utilizes phage amplification or genetically modified phages (e.g., carrying reported genes). These methods already showed some ultrasensitivity but required long incubation time as they rely strongly on the metabolism of the bacteria.

We compared very recent developments described in this review in detail, with best performing phage-based biosensors reported before 2017 (see Table 1 and Figure 3) [13]. The progress is visible, but there is still a definite uncharted territory for bacteria sensing at a concentration below 10 CFU/mL in less than one hour. To the best of our knowledge, the only sensor meeting such requirements were reported in 2020 by Farooq [26]. They used phages deposited on the surface, and bacteria capturing was detected employing differential pulse voltammetry. Next, LOD in the range of 1 CFU/100 mL, also in the time shorter than one hour, should be realized.

## 5. Future of Phage-Based Biosensing

In the last three years, presented developments create an impression that phage-based bacteria detection is a well-established area in practical clinical diagnostics. In reality, however, there are just a handful of companies actively working on such technologies and only one (to our best knowledge) product already available on the Sample6 DETECT HT System (Microbiologique, Seattle, WA, USA). Such drastic and surprising disparity is even more puzzling when one realizes that first phage-based methods reaching the limit of detection of 1 CFU per 100 mL were reported already 17 years ago [30].

As it is continuously repeated in published works, current methods of detecting and identifying bacteria do not meet the requirements in several fields, e.g., healthcare, food industry, or biosafety. The main concern is the time of analysis, which for traditional microbiological methods can extend up to 72 h. This time is too long when a patient’s life is endangered or in case of products with a short expiry date. Several less time-demanding methods were developed, such as PCR, immunomethods, mass spectrometry, spectroscopy (e.g., SERS), and, discussed herein, phage-based approaches. All of them provide faster detection times ranging from a few minutes to a few hours. However, in most diagnostic laboratories around the world, slow and tedious microbiological methods are still used. Thus, the time of detection is not the only important aspect. A broader and more in-depth analysis is required to understand this issue.

Another crucial aspect of any analytical method is the limit of detection (LOD). Microbiological methods provide the highest available LOD of single CFU/L. Such a low limit is needed in particular applications (e.g., potable water), and there are myriad examples where such a good LOD is not necessary. In cases of heavily infected patients or environmental water reservoirs, concentrations of bacteria can reach even 10^3^ CFU/mL [102,103]. In Table 1, it is clear that phage-based methods have LOD allowing for many practical applications; thus, this cannot be the only limiting aspect.

We also indicate the need to demonstrate the possibility of future biosensors to be more easily adaptable for the detection of a variety of target bacteria by merely changing the phage within the sensing element. The focus of the community is on the realization of new designs of sensing devices and methods. To demonstrate the promising features (e.g., time of analysis, LOD), scientists often use a well-known phage-bacteria pair as a model system. There are only a few examples which show the utilization of more than one phage to detect various target bacteria using the same design of the biosensor. For instance, Anany et al. [87] demonstrated three different (rV5, AG2A, CGG4-1) phages for qPCR detection of bacteria. In all three cases, the authors obtained similar LOD in the range from 10 to 50 CFU/mL. Another means of broadening the spectrum of bacteria detected by a given biosensor is to utilize phage cocktails, similar to phage therapies.

The critical factor allowing for the product to reach the market is an effective transition from science to industry. The abovementioned time of analysis and limit of detection are core parameters of any analytical method, but they do not determine successful science–industry transfer. Crucial aspects are, among others, repeatability, stability, portability, ease of use, selectivity, price, and ease of shipping. These parameters are often omitted in scientific work, as a single team typically does most experiments with a unique set of equipment in the laboratory’s stable conditions. The development of all these additional aspects is not required for successful publication but is essential for transforming laboratory experiments to practical analytical techniques. Rarely any effort is made to obtain such improvements, as they are not considered scientific anymore, but are somewhat further engineering. Thus, very few ideas reported in scientific publications are ready for commercialization, and many of them require additional, often expensive development.

Are we there yet with technologies based on bacteriophages? After all, dozens of publications report fast, cheap, and sensitive phage-based bacteria detection spans for over a few decades. Unfortunately, as with developing technology, the transition between scientific publications and widely available products is tedious. With products like Sample6, we indeed started this journey, and many published methods promise to repeat its success.

## Figures and Tables

**Table 1:** Summary of recent developments in phage-based sensors for bacteria detection.

Bioreceptor	Bacteria	Method	LOD	Time	Comments	Reference
**Phages at the surface**						
T4 phage	*Escherichia coli* BL21 DE3	microscopic	10^2^–10^3^ CFU/mL	15 min of incubation	virions properly oriented in the constant electric field	[57]
T2 phage	*Escherichia coli* B ATCC 11303	electrochemical impedance spectroscopy	10^3^ CFU/mL	30 min	virions correctly oriented according to charge driven assembly on carbon nanotube-based impedimetric biosensor	[32]
T4 phage	*Escherichia coli* BL21 DE3	microscopic	10^2^ CFU/mL	15 min of incubation	virions oriented correctly in the alternating electric field	[60]
T4 phage	*Escherichia coli* B	differential pulse voltammetry	14 ± 5 CFU/mL	20 min	virions properly oriented in the alternating electric field on the micro-electrochemical sensor	[36]
lytic phage isolated from the hospital sewage water	*Staphylococcus aureus* CCTCC AB2013186	differential pulse voltammetry	3 CFU/mL in PBS	30 min	the best balance between LOD and time of analysis reported to date	[26]
			5 CFU/mL in milk			
T4 phage	*Escherichia coli* B, ATCC 11303, *Escherichia coli* XLMRF	SERS	1.5 × 10^2^ CFU/mL	10 min of incubation	reusable biosensor	[49]
*Tbilisi* bacteriophage	*Brucella abortus*	SERS	10^4^ CFU/mL	45 min		[51]
Gamma-phages	*Bacillus thuringiensis*	SERS	10^4^ CFU/mL	45 min	the principal component analysis was used for data processing	[52]
M13 phage	*Escherichia coli* XL1-Blue and K12 strains	electrochemical impedance spectroscopy	14 CFU/mL	30 min of incubation	virions chemisorbed on glassy carbon electrode decorated with gold nanoparticles	[35]
M13 phage displaying specific peptide NRPDSAQFWLHHGGGSC (MSal020417)	*Salmonella* spp.	the capacitive flow injection system	2 × 10^2^ CFU/mL	40 min	reusable (up to 40 times) biosensors; virions immobilized on a polytyramine/gold surface	[96]
PaP1 phage	*Pseudomonas aeruginosa*	electrochemiluminescence	56 CFU/mL	30 min	carboxyl graphene-PaP1 composite was dropped onto the glassy carbon electrode	[41]
C4-22 phage	*Salmonella enterica*	magnetoelastic	7.86 × 10^3^ CFU/mm^2^	2 min of incubation	bacteria were captured from the surface of raw chicken breast filet	[44]
phage 12600	methicillin-resistant *Staphylococcus aureus*	magnetoelastic	3 × 10^3^ CFU/mL	30 min	the method is based on sensors previously reported by the same group [42]	[43]
E2 phage	*Salmonella Typhimurium* ATCC 1331	magnetoelastic	5 × 10^2^ CFU/mL	30 min	bacteria were captured from the surface of romaine lettuce	[45]
**Bacteriophage based bioconjugates**						
S13 phage	*Staphylococcus aureus* SA27	dark field microscopy	8 × 10^4^ CFU/mL	15–20 min	virions were oriented according to charge driven assembly on the surface of core−shell nanoparticles	[66]
P9b phage displaying the specific peptide (QRKLAAKLT)	*Pseudomonas aeruginosa* ATCC 27853	SERS	NA	around 2h	gold nanoparticles were used	[64]
chemically modified and genetically engineered M13 phages	*Escherichia coli* (2 strains), *Pseudomonas aeruginosa*, *Vibrio cholerae*, *Xanthomonas campestris* (2 strains)	colorimetric sensor	60 to 10^2^ cell/mL	30 min	gold nanoparticles were used	[65]
T4 phage	*Escherichia coli* BL21	flow cytometry	10^4^ CFU/mL	15 min of incubation	magnetic and fluorescent particles were used	[67]
P-S. aureus-9, isolated from an environmental water sample	*Staphylococcus aureus* (18 clinical strains)	isolation and separation by magnetic bioconjugates + immunoassay	2.47 × 10^3^ CFU/mL in PBS	90 min	no pre-enrichment	[27]
			8.9 × 10^3^ CFU/mL in juice			
temperate phages isolated from environment samples	*Staphylococcus arlettae*	fluorescence quenching	10^2^ CFU/mL	20 min of incubation	IRMOF-3 was used	[28]
	*Staphylococcus aureus*	fluorescence quenching	31 CFU/mL	20 min of incubation	NH_2_-MIL-53(Fe) was used	[29]
T4 phage	*Escherichia coli* ATCC 11303	differential pulse voltammetry	1 CFU/mL	140 min	Cu_3_(PO_4_)_2_ nanoflowers loaded with glucose oxidase, horseradish peroxidase, thionine, and gold nanoparticles to which virions attached were used as the electrochemical signal amplification system	[34]
**Genetically modified phages**						
T7-ALP phage	*Escherichia coli* BL21	fluorescence imaging and image analysis	around 10^2^ bacteria per gram of sample	6 h	fluorescent substrate for alkaline phosphatase activity was added; detection in model beverage samples	[73]
A511::luxAB	*Listeria monocytogenes* WSLC 1001, ScottA, EGDe, *Listeria innocua* WSLC 2012, *Listeria ivanovii* WSLC 3009	magnetic separation combined with fluorescence	around 10^2^ cells/mL	6 h	magnetic beads with cell wall-binding domains from *Listeria* phage endolysins were used for magnetic separation	[74]
NRGp6 phage (T7 with NanoLuc luciferase expression cassette	*Escherichia coli* BL21	spectroscopic detection	5 × 10^2^ CFU/mL	2 h of incubation	NanoGlo substrate was added	[75]
T7 phage with luciferase or an alkaline phosphatase fused with CBM	*Escherichia coli*	visualization of colonies	1 CFU/100 mL	10 h	filtration based method; enzymatic substrate was added	[76]
T7 phage with NanoLuc-CBM	*Escherichia coli*	luminescence of cellulose bound fused proteins	<10 CFU/mL	2.5 h	NanoGlo substrate was added	[77]
T7 phage with NanoLuc-CBM	*Escherichia coli* ECOR13	luminescence	20 CFU/100 mL	5 h	NanoGlo substrate was added	[78]
phiV10lux phage	*Escherichia coli* O157:H7	bioluminescent intensity	around 1 CFU/mL	40 min after 5 h of incubation	LOD in artificially contaminated romaine lettuce 10 CFU/cm^2^, apple juice 13 CFU/mL, ground beef 17 CFU/g	[81]
T7lacZ	*Escherichia coli*	differential pulse voltammetry	10^2^ CFU/mL	7 h	4-aminophenyl-β-galactopyranoside was added as a substrate for β-galactosidase	[33]
*mCherry_bomb_*φ	*Mycobacterium tuberculosis*	fluorescence microscopy	NA	at least 48 h to 96 h	the method allowed for a phenotypic determination of rifampicin resistance; sputum samples were collected from patients	[82]
**Phage amplification**						
p53 phage	*Acinetobacter baumannii* (15 various clinical isolates)	qPCR	10^2^ CFU/mL in serum	4 h		[83]
p53 phage	*Acinetobacter baumannii*	qPCR	10 CFU/mL sputum samples	6 h		[84]
vB_SenS_PVP-SE2 phage	*Salmonella Enteritidis*	qPCR	8 CFU/25 g in chicken samples	10 h		[85]
brucellaphage	*Brucella abortus*	qPCR	1 CFU/mL	72 h	in mixed cultures and blood samples	[86]
rV5 phage	*Escherichia coli* O157:H7	qPCR, phages printed onto paper strips using modified inkjet	10–50 CFU/mL	8 h	in spinach and broth	[87]
AG2A phage	*Escherichia coli* O45:H2				in ground beef	
CGG4-1 phage	*Salmonella* Newport				in chicken samples	
MS2 phage	*Escherichia coli* C-3000	bead-based sandwich-type immunoassay	10^2^ cells/mL	3 h		[88]
**Detection of bacterial metabolites**						
T7 phage	*Escherichia coli* BL21	fluorescence	10 CFU/mL in simulated spinach wash	8 h	resorufin β -D-galactopyranoside was added after lysis	[90]
PAP1 phage	*Pseudomonas aeruginosa*	luminescence	2 × 10^2^ CFU/mL	2 h	firefly luciferase-adenosine triphosphate bioluminescence system was used	[91]
**Phage fragments**						
pVIII protein	*Staphylococcus aureus*	magnetophoretic chromatography in the external magnetic field combined with colorimetric readout due to enzymatic activity of nanozyme	8 CFU/mL	NA	magnetic nanozyme Co_3_O_4_ MNE@fusion-pVIII was used	[95]
cell-binding domain (CBD)	methicillin-resistant *Staphylococcus aureus* (6 strains)	flow cytometry	40 CFU/mL	Around 1 h (2x 30 min incubation + washing steps)	The CBD-GFP fusion protein was used, broad host recognition due to CBD; no lysis	[93]
bacteriophage endolysin CTP1L	*Clostridium tyrobutyricum* (17 strains)	fluorescence microscopy	3 spores per g of cheese	around 35 min + washing steps	GFP-CTP1L and GFP-CBD were used; also bind to clostridial spores	[94]
fiber protein (P069)	*Pseudomonas aeruginosa* (4 strains)	bioluminescence	6.7 × 10^2^ CFU/mL	around 60 min	two very different detection approaches. BL based on magnetic beads, FL on the interactions with modified surface	[92]
		fluorescence	1.7 × 10^2^ CFU/mL	around 80 min		
**Best performing phage-based methods reported before 2017 [13]**						
Lambda phage	*Escherichia coli*	amperometric	1 CFU/100 mL	6–8 h	detection of metabolites	[30]
P22 phage	*Salmonella*	colorimetric	1 CFU/24 mL	6 h	phagomagnetic separation of bacteria labeled with antibodies conjugated with horseradish peroxide	[97]
AR1 phage	*Escherichia coli*	plaque count method	1 CFU/mL	3 h	phage amplification	[98]
PP01 phage	*Escherichia coli*	fluorescence	1 CFU/mL	3 h	genetically modified phages	[99]
M13 phage	*Escherichia coli*	amperometric	1 CFU/mL	3 h	detection of metabolites	[31]
HK620 phage	*Escherichia coli*	flow cytometry	10 CFU/mL	1 h	genetically modified phages	[79]
P22 phage	*Salmonella*					
T7 phage	*Escherichia coli*	flow cytometry	10 CFU/mL	1 h	conjugates of biotinylated phages and streptavidin bound quantum dots	[100]
